# Preclinical and Clinical Development of Noncoding RNA Therapeutics for Cardiovascular Disease

**DOI:** 10.1161/CIRCRESAHA.119.315856

**Published:** 2020-02-27

**Authors:** Cheng-Kai Huang, Sabine Kafert-Kasting, Thomas Thum

**Affiliations:** 1 From the Institute of Molecular and Translational Therapeutic Strategies (C.-K.H., S.K.-K., T.T.), Hannover Medical School, Germany.; 2 REBIRTH Center of Translational Regenerative Medicine (T.T.), Hannover Medical School, Germany.

**Keywords:** animal models, cardiovascular diseases, nucleic acids, nucleosides, nucleotides, therapeutics

## Abstract

RNA modulation has become a promising therapeutic approach for the treatment of several types of disease. The emerging field of noncoding RNA-based therapies has now come to the attention of cardiovascular research, in which it could provide valuable advancements in comparison to current pharmacotherapy such as small molecule drugs or antibodies. In this review, we focus on noncoding RNA-based studies conducted mainly in large-animal models, including pigs, rabbits, dogs, and nonhuman primates. The obstacles and promises of targeting long noncoding RNAs and circRNAs as therapeutic modalities in humans are specifically discussed. We also describe novel ex vivo methods based on human cells and tissues, such as engineered heart tissues and living myocardial slices that could help bridging the gap between in vivo models and clinical applications in the future. Finally, we summarize antisense oligonucleotide drugs that have already been approved by the Food and Drug Administration for targeting mRNAs and discuss the progress of noncoding RNA-based drugs in clinical trials. Additional factors, such as drug chemistry, drug formulations, different routes of administration, and the advantages of RNA-based drugs, are also included in the present review. Recently, first therapeutic miRNA-based inhibitory strategies have been tested in heart failure patients as well as healthy volunteers to study effects on wound healing (NCT04045405; NCT03603431). In summary, a combination of novel therapeutic RNA targets, large-animal models, ex vivo studies with human cells/tissues, and new delivery techniques will likely lead to significant progress in the development of noncoding RNA-based next-generation therapeutics for cardiovascular disease.

It is well known that <2% of the human transcriptome encodes protein-coding RNAs, whereas the majority are noncoding RNAs (ncRNAs), including ribosomal RNA, tRNA, microRNA (miRNA, or miR), long noncoding RNA (lncRNA), circular RNA (circRNA), and other small RNAs.^1,2^ Over the past 2 decades, there has been increasing evidence that ncRNAs act as key players in the onset and progression of cardiovascular diseases (CVDs).^3–5^ As the ncRNA research field has progressed, researchers have developed complex tools to modulate these ncRNAs with the aim of establishing novel, next-generation strategies to combat CVDs.^6^ For example, some of the first miRNA or lncRNA targets identified in cardiac remodeling were miR-21 and the lncRNA *Chast*.^7,8^ Therefore, ncRNA-orientated next-generation drugs might offer a novel therapeutic option for CVDs, for which innovations have been scarce in the last few decades.

CVDs are the main cause of death in both Europe and the United States, according to Atlas (European Society of Cardiology) and the Centers for Disease Control and Prevention (CDC, United States).^9,10^ One of the drawbacks to develop new therapeutic innovations is that most observations have only been made in in vitro systems or small animal models (eg, rodents) but have not yet been replicated or have failed to be replicated in larger animal models. Indeed, rodents exhibit several fundamental differences in certain cardiovascular physiology elements, such as heart weight, heart rate, blood pressure, and the coronary artery system, in comparison to larger animals and humans that may influence experimental conclusions.^11,12^ Excitingly, several RNA-based drugs targeting CVD have already been approved by the US Food and Drug Administration, and the pharmaceutical development of ncRNAs is also currently under way.^13–15^ To progress in clinical application, proof of concept and safety evaluation in large animal models, such as pigs and nonhuman primates, are helpful and often necessary steps before the start of first-in-human trials. Thus, in the present review, we focus on ncRNA-targeting therapeutic studies mainly performed in large animals and current clinical trials.

## MiRNA Studies in Large Animals

**Figure 1.**
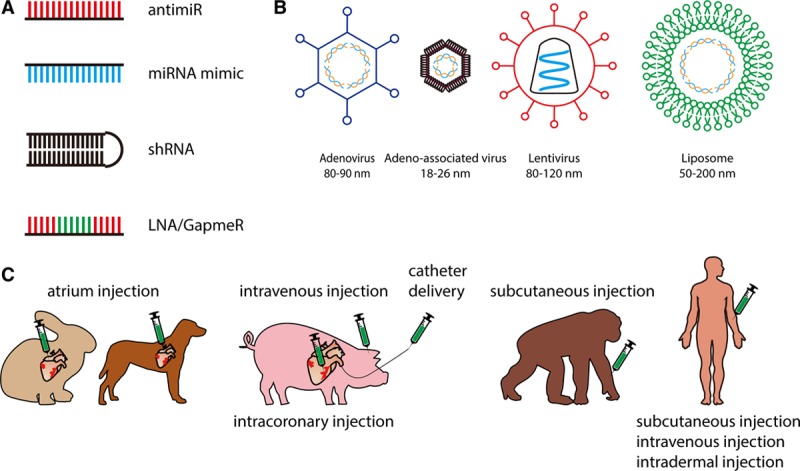
**Scheme of oligonucleotide-based RNA delivery.** **A**, AntimiRs (miRNA inhibitors) can be modified with different chemical modifications, including locked nucleic acids (LNAs) and sugar backbone modifications (2’-O-Me, 2’-F/MOE, and 2’-O-MOE), while miRNA can also be enhanced via miRNA mimics. To inhibit mRNAs or long noncoding RNAs (lncRNAs), short hairpin RNAs (shRNAs), or LNA/GapmeR are commonly used. **B**, Adenovirus, adenoassociated virus (AAV), and lentivirus particles can be used as a vector to silence or overexpress target genes. In addition to viral-based delivery, liposomes or nanoparticles are another way through which to deliver antimiRs or miRNA mimics. **C**, Various delivery approaches can be applied in different species. For example, atrium injection is performed in rabbits and dogs with atrial fibrillation. For pigs, intravenous injection, catheter-based injection, and intracoronary injection are commonly used. Subcutaneous injection can be also used. Clinically, subcutaneous injection, intravenous injection, and intradermal injection are more attractive and easier delivery routes in humans.

MiRNAs are a class of ncRNAs with short (≈18–22 nucleotides) and highly conserved sequences that predominantly exist in eukaryotes. Functionally, miRNAs are involved in various gene regulatory mechanisms including mRNA degradation and translational repression via the RNA-induced silencing complex.^16^ Since miRNAs are highly conserved, exhibit short sequences, and are highly abundant, they became the first class of ncRNAs studied in large animal models (Tables 1 and 2; Figure 1) and, recently, clinical trials (Table 3; Figure 1).

**Table 1.**
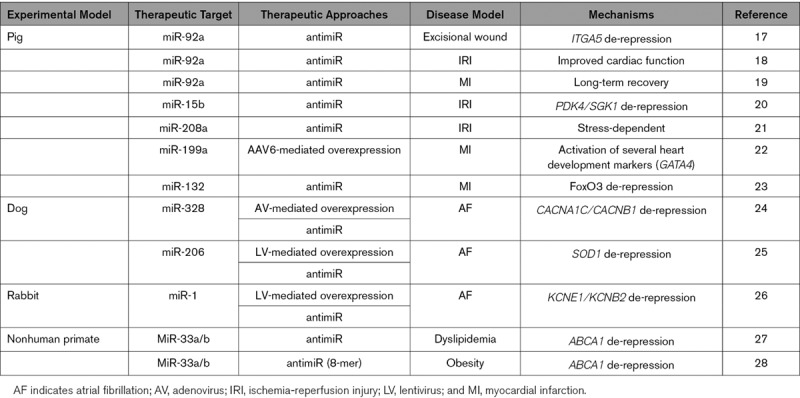
Modulation of miRNA Expression in Different Large Animal Models

**Table 2.**
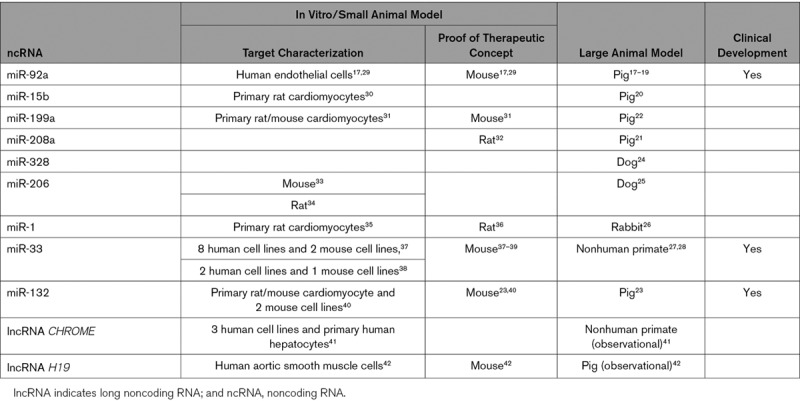
The Developmental Progression of ncRNA Studies in Different Models and Clinical Trials

**Table 3.**
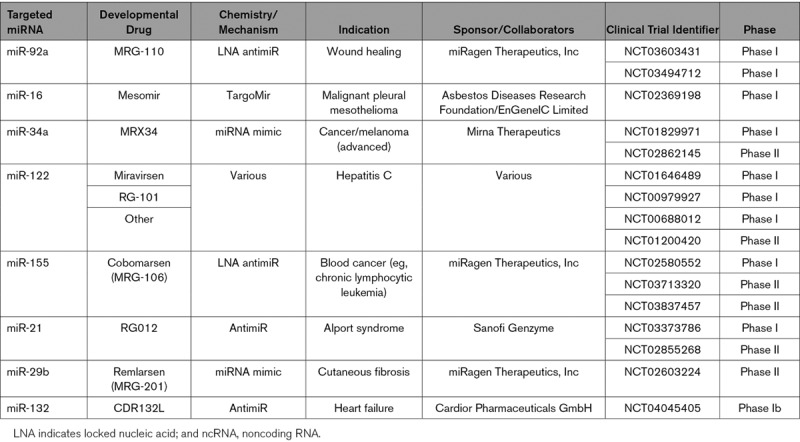
Clinical Trials With ncRNA-Based Therapeutics

### Pig Studies

Pigs are a popular model for mimicking human heart disease, especially for myocardial infarction (MI) studies, since the porcine heart shares many similarities with the human heart, including heart weight, blood pressure, and heart rate.^11,43,44^

One of the first studies that investigated miRNA therapeutics in large animal models targeted the miRNA miR-92a. MiR-92a is ubiquitously expressed and has multiple functions in the body, including the modulation of angiogenic pathways.^29^ In a mouse model, it was shown that miR-92a was upregulated after cardiac ischemic injury. Silencing miR-92a by 2’-O-methyl (2’-O-Me)-modified antagomir-92a significantly enhanced angiogenesis in vitro and in vivo. Furthermore, the inhibition of miR-92a in a MI mouse model reduced the infarct size and improved certain cardiac functions.^29^ Meanwhile, in an ischemia-reperfusion injury pig model, there was a reduction in infarct size, less cardiomyocyte apoptosis, and better myocardial function after the inhibition of miR-92a expression.^18^ The downregulation of miR-92a also increased capillary density and reduced cardiac inflammation; however, this study focused only on the short-term (three or seven days) effect of antagomir-92a treatment. To study more long-term effects and overcome the potential off-target issues of a systemic miR-92a blockade, Bellera et al^19^ delivered antimiR-92a encapsulated in bioabsorbable and biocompatible microspheres via intracoronary injections in a MI pig model. The microsphere-antimiR-92a was detected mainly in the capillaries of the anterior myocardial wall and surprisingly showed no distribution to remote organs. Regarding the long-term effects of microsphere-antimiR-92, the treatment also induced angiogenesis 1 month following MI induction. This data revealed that a drug meant to inhibit miRNAs may have higher specificity and a greater long-term effect when modified with proper physical protections or conjugation chemistries. A miR-92a inhibitor was further tested in 2 phase I clinical trials (Table 3) and was named MRG-110 (miRagen Therapeutics, Inc, NCT03603431 and NCT03494712).^17^ MRG-110 is expected to accelerate wound healing by improving blood flow via its proangiogenic properties. Indeed, Gallant-Behm et al^17^ demonstrated in a pig model that the administration of antimiR-92a inhibitors significantly increased blood flow and revascularization in peri-wound areas. The results of these phase 1 studies have not yet been published.

Another mechanism regulated by miRNAs and often contributing to CVD is mitochondrial dysfunction.^45^ MiR-15b has been shown to be involved in mitochondrial dysfunction by targeting Arl2 (ADP-ribosylation factor-like 2). In a primary rat cardiomyocyte model, both cellular atrial tachypacing (ATP) levels and Arl2 mRNA expression decreased following miR-15b overexpression, while miR-15b inhibition reversed this phenotype.^30^ Hullinger et al^20^ further applied locked nucleic acid (LNA)-modified antimiR-15b to a MI pig model and showed that miR-15b inhibition restored porcine cardiac function. In addition to a 16-mer antimiR, researchers also developed a short 8-mer antimiR-15b and found that it efficiently suppressed miR-15b expression and also enhanced cardiac function. Interestingly, there were differences between the 2 oligonucleotide inhibitors. For example, treatment with a 16-mer (but not an 8-mer) antimiR increased left ventricular end-diastolic pressure, whereas treatment with only the 8-mer antimiR significantly reduced infarct size.^20^ These data indicated the importance of designing miRNA inhibitors to achieve an efficient therapeutic response.

Importantly, the pharmacological effects of antimiRs might be influenced by the disease condition. For instance, the cardiac-enriched miR-208a is encoded from the intron of the *α-MHC* gene and has been reported to be responsible for cardiac hypertrophy and fibrosis.^46^ Montgomery et al^32^ further demonstrated that the inhibition of miR-208a improved cardiac function in a hypertension-induced heart failure rat model. Eding et al^21^, however, showed that differentially expressed downstream genes modulated by antimiR-208a are different in TAC and MI rat models, and a similar stress-dependent antimiR effect was also observed in a pig MI model. These results, therefore, suggested that the disease type and severity of a disease should be considered in the preclinical development of a miRNA drug.

Another miRNA, miR-132, was shown to be crucially involved in cardiac growth and autophagy.^40^ Indeed, miR-132 is both necessary and sufficient for driving pathological cardiomyocyte growth, a hallmark of adverse cardiac remodeling. Recently, the safety, tolerability, favorable pharmacokinetics, dose-dependent pharmacokinetic/pharmacodynamic (PK/PD) relationships, and the high clinical potential of an antimiR-132 treatment in pigs following myocardial infarction has been documented.^23^

It is known that the adult mammalian heart has no significant regenerative capacity following injury, causing massive cardiomyocytes loss and subsequently leading to cardiac dysfunction and heart failure. Based on a whole-genome miRNA library screening that compared postnatal day 1 and day 7 rodent hearts, miR-199a was identified and suggested to promote the cardiomyocyte cell cycle re-entry both in vitro and in vivo. The overexpression of miR-199a increased cardiomyocyte proliferation and preserved cardiac function after inducing MI in mice.^31^ The same group next overexpressed miR-199a in pigs after MI via the intramyocardial injection of adeno-associated virus-containing miR-199a.^22^ Indeed, the overexpression of miR-199a in pig hearts post-MI improved cardiac contractility, increased muscle mass, and reduced scar size; however, 70% of the adenoassociated virus-miR-199a treated pigs (7 out of 10) died from sudden cardiac death 7 to 8 weeks after virus injection. Further histological analysis revealed that a small group of cells expressing cell proliferation markers (eg, Ki67) and early heart development markers (such as GATA4) were infiltrating the infarcted myocardium. These cells were poorly differentiated, highly proliferating, and immature premyocytes that likely induced the observed ventricular fibrillation and sudden cardiac death of the pigs.^22^ Overall, this miR-199 pig study impressively demonstrated the power of miRNAs in achieving biological effects in the heart and highlighted the need for the careful preclinical characterization and off-target effect prediction of miRNA-based drugs before clinical testing.

Due to the similarity between pigs and humans regarding their cardiovascular systems and physiology, (mini-)pigs can also be valuable models for atherosclerosis. Based on different genetic alterations, minipigs with constitutive and/or diet-dependent increases in serum cholesterol have already been generated and used in drug testing. For instance, strains with an altered LDL receptor gene or apolipoprotein E deficiency had increased serum cholesterol and developed atherosclerosis.^47,48^ The engineered heart tissue (EHT) made from miniature pigs carrying the hypertrophic cardiomyopathy mutation *MYH7 R403Q* has presented increased stiffness and impaired muscle relaxation.^49^ Mentzel et al^50^ investigated the miRNA profiles of diet-based obese minipigs and found several miRNAs to be potential biomarkers and therapeutic targets. In the future, the testing of ncRNA therapeutic efficacy in such disease models may provide important contributions to a mechanistic understanding and pharmaceutical exploitation of the respective RNA compounds.

### Dog and Rabbit Studies

In contrast to pigs, dog hearts have abundant collateral coronary vessels and thus are not easily useable as a MI model.^11,44,51^ In contrast, dog hearts have an electrophysiological system very similar to that of humans, are prone to develop atrial fibrillation (AF), and are thus often used as a preferable model for AF research.

There are a variety of methods to induce AF in dogs, including nicotine treatment and ATP.^24,25,52,53^ In an ATP-induced AF-dog model, miR-328 was found to be upregulated; moreover, the overexpression of miR-328 via an adenoviral approach recapitulated AF phenotypes in healthy dogs. Additionally, computational prediction revealed that the calcium voltage-gated channel subunits α1c and β1 are 2 genes targeted by miR-328. Treatment with antimiR-328 significantly de-repressed the expression of *CACNA1C* and *CACNB1* and reversed AF.^24^

In addition, miR-206 was shown to participate in AF progression. MiR-206 is a muscle-enriched miRNA and is also required for the regeneration of neuromuscular synapses. The knockout of miR-206 in an amyotrophic lateral sclerosis mouse model accelerated the disease progression.^33^ The miRNA profiling in an AF-dog model revealed that miRNA-206 was induced 10-fold compared to in the control group. Additionally, the inhibition of miR-206 by lentiviral-antimiR-206 injection attenuated the AF-induced symptoms.^25^ Although neuronal regeneration induced by miR-206 indicated the essential role of miR-206 during muscle denervation and reinnervation,^33,34^ the overexpression of miR-206 aggravated the AF-induced symptoms. These results highlight that miRNAs could possess different functions in different organs and sometimes exhibit species-specific effects.

In an ATP-induced AF rabbit model, miR-1 was reported to promote cardiac arrhythmias and enhance calcium release by targeting several ion channel genes. These findings were also observed in mouse and rat models.^26,35,36^ The inhibition of miR-1 via lentiviral-based antimiR-1 infections significantly prolonged the atrial effective refractory period and de-repressed potassium voltage-gated channel (KCN) E1 and B2 expression, 2 target genes of miR-1.^26^

Atherosclerosis studies have been performed in Watanabe heritable hyperlipidemic rabbits since their development/discovery in the 1970s. Meanwhile, 2 advanced strains were generated: one showing spontaneous coronary atherosclerosis (Watanabe heritable hyperlipidemic-CA) alone and the other showing myocardial infarction (Watanabe heritable hyperlipidemic-MI).^54,55^ Despite certain differences from human pathophysiology, these animal models can be useful tools for the investigation of new drug candidates. However, there have so far been no reports on the profiles of the effect of miRNA, other classes of ncRNA, nor their inhibitors in Watanabe rabbits.

### Nonhuman Primate Studies

Chronic heart failure, subacute MI models, as well as models of atherosclerosis have also been studied in nonhuman primates^56,57^; however, due to ethical and financial issues, primates are not frequently used in cardiovascular research.

The transcription factor SREBP (sterol-response element-binding protein) regulates genes involved in cholesterol biosynthesis, such as ABCA1 (ATP-binding cassette transporter A1). A loss of ABCA1 expression can cause Tangier disease, which is characterized by a low level of circulating HDL.^58^ Najafi-Shoushtari et al and Rayner et al showed that the human SREBP genome locus transcribes not only mRNA but also 2 miRNAs, miR-33a and miR-33b. MiR-33 inhibits the expression of ABCA1, which leads to circulating HDL-C reduction and, therefore, the silencing of miR-33 increased HDL-C expression in a mouse model.^37–39^ Despite the promising results of developing miR-33 as a therapeutic target against dyslipidemia and atherosclerosis, its clinical progress is limited. MiR-33b, which is encoded from the *SREBP1* gene locus, only exists in large animals and not in mice. This difference may also significantly affect the results studied using knockout mouse models or insulin response experiments in mice.^59^ To solve this issue, Rayner et al^27^ injected 2’-fluoro/-O-methoxyethyl (2’-F/MOE)-modified antimiR-33a/b subcutaneously to treat African green monkeys (*Chlorocebus aethiops*) with dyslipidemia. They found the same results as observed in the mouse model: the knockdown of miR-33a/b increased ABCA1 expression and plasma HDL-C levels. Interestingly, beyond cholesterol metabolism, they also found genes involved in fatty acid oxidation and biosynthesis to be regulated. These effects resulted in the reduction of plasma VLDL (very low density lipoprotein) triglyceride levels, a new finding that was not observed in the mouse model.^27^

Another study employed subcutaneous administration of short seed-targeting 8-mer antimiRs in obese African green monkeys.^28^ In this study, the de-repression of several miR-33 target genes, including ABCA1m were observed, plasma HDL-C levels were elevated, and no adverse effects were noticed.^28^ These 2 studies performed in nonhuman primates provided evidence that the inhibition of miR-33a/b to raise plasma HDL-C levels could be a promising therapeutic strategy for the treatment of dyslipidemia.

## LncRNA and circRNA Studies in Large Animals

LncRNAs are another class of ncRNAs with longer (>200 nucleotides) but less conserved sequences.^60^ Having various biological functions, lncRNAs are certainly promising therapeutic targets; however, translational studies in animals are difficult with this class of ncRNA due to their poor sequence conservation between species.^61,62^ Thus, only well-conserved lncRNAs seem promising as translationally relevant disease targets for new therapies. Indeed, the number of conserved lncRNAs is still quite limited.^8,63,64^ As the degree of DNA/RNA sequence conservation among different species is commonly used to predict the biological functions of the species,^65,66^ it explains why lncRNA-targeting experiments are not frequently performed in large animals. Studies have begun to identify novel un-annotated lncRNAs in different large animal models. Kern et al analyzed lncRNAs from three farm animals (chicken, cattle, and pigs) and found that half were not annotated in NCBI or other databases. As expected, the lncRNAs from these species were less conserved. Interestingly, researchers also found that many have locus-conserved transcripts (a transcript with a diverged sequence but the same genomic position as its neighboring genes), which might indicate similar biological functions between themselves.^67^ In dogs, Béguec et al analyzed the lncRNA profile of 26 different tissue types and developed a tool called FEELnc.^68^ Surprisingly, around 900 lncRNAs (10%) were highly conserved to human transcripts, including well-known *HOTAIR*, *MALAT1*, and *NEAT1*.^68,69^ In addition to these specific species, large-scale lncRNA analysis in >7 divergent species, from zebrafish to humans, was also reported.^70,71^

The dynamic expression of lncRNAs in the progression of heart disease is also important. In a porcine ischemic heart model, RNA-seq was performed to compare the expression of lncRNAs between healthy and ischemic zones of the heart. Four hundred fifty lncRNAs were identified that were not previously annotated and were differentially regulated after ischemic injury. Among these novel lncRNAs, transcripts that are transcribed antisense to myocardial transcription factors, such as *GATA4*, *GATA6*, and *KLF6*, were identified and observed to potentially have important biological functions in the heart.^72^ An experimentally validated database (a heart disease-related, noncoding RNA database, HDncRNA) developed by Wang et al contains around 2000 lncRNAs that are associated with heart diseases in 6 species, including humans, rodents, pigs, calves, and dogs. This database is equipped with a web-based interface that allows users to easily search for lncRNA candidates, directing them to the original relevant publications.^73^ Recently, Wu et al^74^ analyzed the lncRNA-mRNA network in carotid atherosclerotic rabbit models and discovered several novel lncRNAs involved in the disease progression of atheroscelrosis.

In spite of the obstacles mentioned above, there have been 2 lncRNA studies performed in large animals. LncRNA *CHROME*, identified by Hennessy et al,^41^ was found to be upregulated in nonhuman primates with atherosclerotic vascular disease. Further in vitro data showed that the overexpression of *CHROME* in HepG2 cells reduced miR-33 expression and de-repressed the miR-33-targeted genes, including *ABCA1*, while the inhibition of *CHROME* by shRNA or LNA GapmeR in primary human hepatocytes and HepG2 cells had opposite effects. Likewise, Li et al^42^ demonstrated that the expression of lncRNA *H19* increased in 2 abdominal aortic aneurysm mouse models as well as a low-density lipoprotein receptor (*LDLR*) knockout mini-pig aneurysm model. The in vitro knockdown of *H19* decreased the apoptotic rate of human smooth muscle cells. Overall, further and more therapeutic experiments in large animal models are needed, since no lncRNA therapeutic approach has been performed in large animals thus far.

Despite their low sequence conservation, lncRNAs have higher tissue specificity. According to a study published by Cabili et al,^75^ 78% of lncRNAs may be tissue-specific, which is much higher than the percentage reported for mRNAs (19%). This conclusion is also supported by a recently published large-scale RNA-seq analysis that revealed 51% to 63% of lncRNAs to be tissue specific.^71^ This specificity makes certain conserved lncRNAs promising targets for drug development, since drugs designed to act based on tissue-specific lncRNAs and their interaction may produce less remote off-target effects.

Circular RNAs (circRNAs) are another novel class of RNA molecules, have a structure featuring covalently linked 3’ to 5’ ends,^76^ and are highly abundant in the human genome.^2,77^ A review published recently summarized the role of circRNAs in cardiovascular biology. In this review, Aufiero et al^78^ listed several circRNAs with functions in rodent heart disease models. For example, a circRNA termed heart-related circRNA (HRCR) was reported to have cardioprotective functions via sponging miR-223. The overexpression of *HRCR* inhibited miR-223 activity and de-repressed the downstream protein ARC and therefore attenuated hypertrophic responses.^79^ Hansen et al^80^ reported that a circRNA called *ciRS-7* (currently named *Cdr1as*) could serve as a miRNA sponge and be involved in heart diseases. Later, *Cdr1as* was further proven to promote myocardial infarction by sponging miR-7.^81^ In addition to cardiomyocytes, Garikipati et al demonstrated that the overexpression of *circFndc3b* in endothelial cells enhanced angiogenic activity and reduced endothelial apoptosis. The cardioprotective mechanism of *circFndc3b* was to interact with the RNA-binding protein FUS to regulate the VEGF-A signaling pathway.^82^ Since circRNAs stem from mRNAs, recently, several studies have also reported that lncRNA/circRNA-mRNA-miRNA networks play an important role in heart development and disease, such as AF and atherosclerosis.^83–86^ For example, Zhang et al^86^ found 7 circRNAs that functioned in cell adhesion, cell activation, and the immune response, which provided an overall better understanding of the pathogenesis of atherosclerosis. With the increasing importance of machine learning algorithms and artificial intelligence, we hope that the better interpretation of such network interactions can lead to an improved understanding of ncRNA networks and their effects on diseases. With the help of network prediction, *Cdr1as* was also shown to regulate neuronal activity in brains by forming a specific ncRNA regulatory network together with the lncRNA *Cyrano* and miR-7/miR-671.^87^ High sequence conservation, abundant quantity, and a higher stability than mRNA are all other advantages of circRNA in terms of its potential to be studied in large animal models and its future consideration as a therapeutic target in humans.^88–90^ Several studies have reported that 15% to 30% of circRNAs are conserved between 3 main species: mouse-human, mouse-pig, and pig-human.^91–93^

## Human EHTs and Living Myocardial Slices

**Figure 2.**
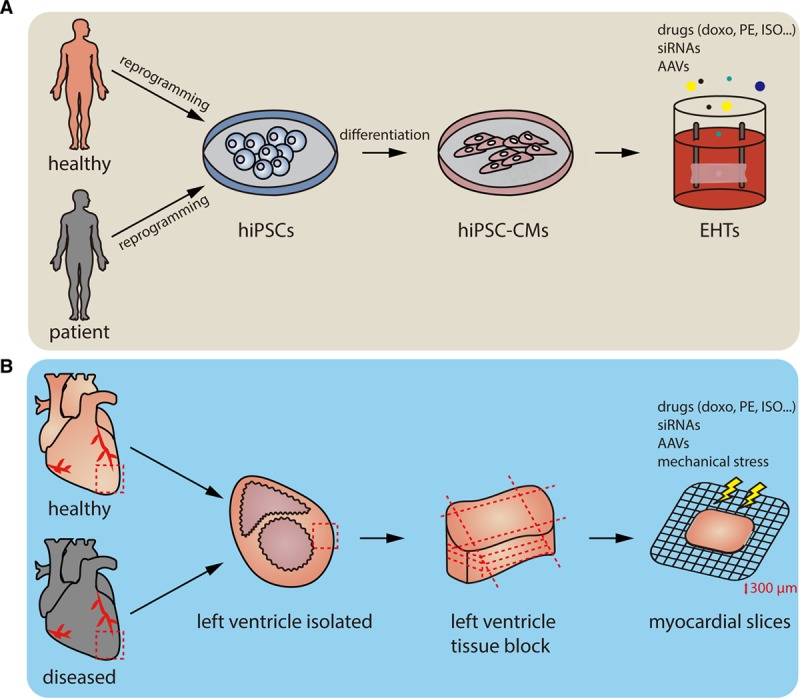
**Workflow of 2 3-dimensional ex vivo models, engineered heart tissues (EHTs), and living myocardial slices.** **A**, Somatic cells are isolated from human blood cells or skin cells, reprogrammed into human induced pluripotent stem cells (hiPSCs), and differentiated into hiPSC-derived cardiomyocytes (hiPSC-CMs). The hiPSC-CMs are seeded onto the scaffolds to generate beating EHTs. Compared with a 2-dimensional cell culture system, EHTs exhibit a better structure and matured phenotypes that are similar to adult CMs. Through modifying the stiffness of the scaffold, different disease models, such as hypertrophic cardiomyopathy, can be established. EHTs can further be tested for drugs or as gene modulation tools. Not only stemming from healthy humans, EHTs can also be made from patients suffering from heart disease for disease modeling. **B**, To prepare living myocardial slices, small or large mammalian hearts, including human hearts, are explanted. The left ventricles or other parts of the heart are then isolated and dissected into small tissue blocks. Hundred to four hundred micrometers myocardial slices are sliced and used for further functional studies, for example, by treating with different drugs or adjusting the voltage that stimulates the contraction of the myocardial slices. Similar to EHTs, the living myocardial slices could also be prepared from diseased animal models. Doxo indicates doxorubicin; ISO, isoproterenol; and PE, phenylephrine.

Although data from large animal models may be more predictive for human cardiovascular diseases, these studies also continue to possess certain limitations. For example, large animals require larger breeding space and higher maintenance costs, and experimental interventions may be time-consuming and do not allow for high repetition. Such circumstance makes it difficult for researchers to collect enough samples in a reasonable time to achieve statistical significance.^12,94^ In addition, large animals have longer gestation times, which makes it difficult to generate gene knockout/knockin models, although the recent emergence of the CRISPR/Cas9 (clustered regularly interspaced short palindromic repeats/Cas9) technique may help solve this problem.^11,95^ Due to the limitations mentioned above, EHT and living myocardial slice models derived from human cells or tissues may serve as a bridge between in vitro and in vivo models (Figure 2).^96,97^

Since the generation of human-induced pluripotent stem cells (hiPSCs) was reported,^98,99^ studies on the differentiation of hiPSCs into various functional cell types, including cardiomyocytes, have rapidly increased in number.^100,101^ However, hiPSC-derived cardiomyocytes (hiPSC-CMs) cultured in monolayer systems show immature and fetal phenotypes that do not reflect the adult heart and fail to recapitulate chronic heart disease phenotypes.^102^ EHTs composed of hiPSC-CMs and additional supporting cells in a 3-dimensional culture system may better reflect a fully developed heart under corresponding disease models.^103,104^ The EHT, sometimes mixed with fibroblast or endothelial cells, has shown improved adult phenotypes, including rod-shaped cardiomyocytes with well-organized sarcomere structures, systolic contraction, and inotropic responses to drug stimulation.^105,106^ Tiburcy et al^105^ further treated isoprenaline, a β1- and β2-adrenoceptor agonist to hiPSC-CM EHTs, to mimic hypertrophic responses, which demonstrated the possibility of using EHT as heart failure and cardiac repair models. HiPSCs can not only be generated from healthy individuals but also from patients who suffering from heart disease. Prondzynski et al^107^ generated EHTs from hypertrophic cardiomyopathy patient-derived hiPSC-CMs, and the hypertrophic cardiomyopathy-EHTs showed phenotypes including cardiac hypertrophy, hypercontractility, and higher myofilament calcium sensitivity. The overall results exhibited the possibility of using EHTs in personalized medicine approaches in the near future.

Recently, living myocardial slice technology has emerged as another option for further experimental evaluation before and in addition to large animal models or clinical trials. Here, cardiac tissue is cut into thin slices by a vibratome, and such slices provide a 3-dimensional structure containing various cell types and exhibit preserved electrical and mechanical connection. This technology has proven to be a platform for studying electrophysiology, drug screening, cardiac fibrosis, and heart failure in cardiac slices that are obtained from several animals, including rats, guinea pigs, rabbits, dogs and, recently, also humans.^108–113^ Watson et al described a detailed protocol for the preparation of adult ventricular myocardial slices with preserved cardiomyocyte viability (97%) and functionality for up to 1 week. The thickness of each myocardial slice is 100 to 400 µm, which allowed for oxygen and small compounds to diffuse through the slice. Moreover, ultrathin slices also make it possible to produce many experiments from the same heart and therefore reduce the number of animals needed in a study.^97,114^

## Clinical Experiences With Coding and Noncoding RNA Therapeutics

Since the biological relevance of ncRNAs has been recognized, the cardiovascular community has begun to develop modulators of these targets as a new generation of cardiovascular therapeutics. In fact, RNA-based therapeutics were first developed in the 1990s, and the first Food and Drug Administration-approved RNA-based drug dates back to 1998, when a 21-mer phosphorothionate oligonucleotide (fomivirsen) targeting CMV IE-2 protein received Food and Drug Administration approval.^14^ Since then, 6 more compounds have been approved by the Food and Drug Administration based on an anti-RNA mechanism targeting mRNAs relevant to age-related macular degeneration, neuromuscular disorders, familial hypercholesterolemia, and transthyretin-mediated amyloidosis, which is involved in heart failure due to the cardiac deposition of TTR amyloid fibrils.^115^ Thus, almost 50% of these innovative drugs focused on indications in the cardiovascular field (Table 4). However, Mipomersen (a GapmeR targeting Apolipoprotein B-100) is no longer marketed in the United States, and 2 recently approved drugs for the treatment of transthyretin-mediated amyloidosis still need to exhibit clinical success in a competitive market, in which the high costs of treatment could be a major drawback.^116^

**Table 4.**
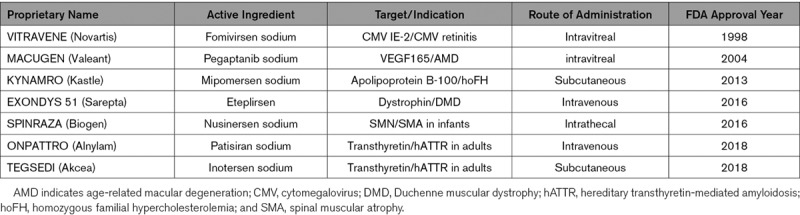
FDA-Approved Antisense Drugs

Current clinical antisense-based drug developments are numerous (recently reviewed by Bennet et al^13^) and include CVD targets such as PCSK9 in LDL-C-hypercholesterolemia (NCT01350960, NCT02597127), Apolipoprotein CIII in familial chylomicronemia syndrome (Volanesorsen received conditional marketing approval in the EU; available via the Early Access Program in the US, NCT 03544060), or Lipoprotein A (Novartis/Akcea Therapeutics, NCT04023552; Amgen, NCT03626662). Moreover, further drugs are currently in a developmental pipeline for the treatment of CVD, targeting mRNAs for angiopoietin 3, factor XI, or apolipoprotein(a), among others.^117–119^ All these aforementioned therapeutic entities use an RNAseH-dependent mechanism or siRNA/RNAi to repress the expression levels of the target transcript. Other drugs targeting mRNAs make use of splicing modulation to improve the expression of a beneficial functional transcript over an altered or missing splicing product in the relevant disease, such as dystrophin in Duchenne muscular dystrophy or the SMN protein in spinal muscular atrophy (see Eteplirsen and Nusinersen in Table 4). Splicing modulation will, in principle, be a relevant mechanism for lncRNAs and circRNAs upon their being targeted as pharmaceutical agents in the future. As described above, these mechanisms are currently subjects of intensive research.

MiRNAs have so far reached the clinical stage, although clinical studies using or targeting miRNAs are still more scarce than antisense strategies for mRNAs. The majority of results from the US database of clinical trials (www.clinicaltrials.gov) refer to the evaluation of miRNA as biomarkers or prognostic factors. Still, a number of miRNAs are currently under clinical development and are summarized below (Table 3).

### Organ Fibrosis

A compound mimicking miRNA-29a in clinical development aims to increase the functional levels of miRNA-29a to combat fibrosis. MiRNA-29a has been shown to reduce collagen expression and is downregulated in multiple fibrotic conditions, including, but not limited to, fibrosis of the heart, lungs, liver, and kidneys and systemic sclerosis.^120^ One early comprehensive study revealed that miR-29a plays an important role in the pathological remodeling of the heart after myocardial infarction.^121^ Recently, and in contrast to the proposed beneficial effects of miR-29a overexpression, it has been demonstrated that cardiomyocyte-expressed miR-29 promotes pathological remodeling of the heart by activating Wnt signaling.^122^ MiRNA-29a mimic, called Remlarsen (MRG-201),^123^ was successfully tested in a phase I study with drug administration to 54 healthy volunteers (NCT02603224); currently, a phase II clinical trial targeting cutaneous fibrosis is being conducted to determine if the substance can limit the formation of fibrous scar tissue in certain skin diseases (NCT03601052). These studies could pave the way toward the investigation of this drug in idiopathic pulmonary fibrosis and other conditions of pathological fibrosis.

MiR-21 is a profibrotic molecule discovered in 2008 that is currently being targeted in a clinical phase II trial. AntimiR-21 has been described as strongly antifibrotic^7^ and is currently clinically developed for the treatment of Alport syndrome, a collagen IV defect causing fibrotic kidney disease, hearing loss, and eyesight problems.^124–126^ A natural history study and a first-in-man trial have both been successfully completed (NCT03373786). A phase II trial for the assessment of safety, tolerability, and efficacy in reducing the decline in renal function has been initiated in a randomized, double-blind, placebo-controlled design, with weekly subcutaneous injections of either the test substance or a placebo over 48 weeks (NCT02855268).

### Ischemic Conditions and Heart Failure

Another compound intended to promote the growth of new blood vessels by inhibiting miR-92a (MRG-110) is currently under clinical development. The beneficial effects of miR-92a silencing in ischemic heart conditions and for the promotion of angiogenesis, as observed in mice and pigs, have been described above. A phase I trial for the investigation of an intradermal injection of miR-92 antimiR in wound healing and incisional complications recently completed recruitment (NCT03603431). The safety of antimiR-92 administration via intravenous injection has been assessed in healthy volunteers, but the results of the study are not yet publicly available (NCT03494712).

Recently, a clinical dose ascending and dose repetition phase 1b study was initiated to assess the safety, pharmacokinetics, and pharmacodynamic parameters of an antimiR-132 inhibitor in stable heart failure patients (NCT04045405). Preclinical data suggested this miRNA plays a key role in the pathological cardiac remodeling process.^23,40^

### Other Clinical Developments

Another inhibitor against a miRNA, miR-155, previously also described in cardiovascular disease,^127^ is being developed for the treatment of various blood cancers, including, but not limited to, T-cell lymphoma and chronic lymphocytic leukemia. This LNA antimiR, called Cobomarsen (MRG-106), has reached clinical phase II with 2 currently active trials, PRISM and SOLAR (NCT03713320, NCT03837457). Two other developments rely on increasing the function of miR-16 (NCT02369198) and miR-34a (NCT01829971) in patients with various advanced malignancies. A phase I trial using TargomiRs (minicells targeted to EGFR) loaded with miR-16-based mimic microRNA was completed with encouraging results.^128^ However, the effects of TargomiRs in patients with malignant pleural mesothelioma require further investigation. One phase I trial with a miR-34a mimic (MRX34) enrolling 155 subjects was withdrawn by the sponsor after 5 serious immune-related adverse events (NCT01829971). This illustrates the potential immunogenicity and off-target effects induced by some RNA drugs.^129^

An antimiR-122 has been evaluated for the treatment of hepatitis C in patients who did not respond to pegylated-interferon alpha and ribavirin; however, its clinical development has so far not proceeded beyond phase II (NCT02508090, NCT02452814).

## Drug Formulations and Different Routes of Administration

As mentioned above, ncRNA-based therapies have recently attracted increasing attention. Compared with other drug formulations, like small molecules or antibodies, RNA therapies have several advantages. Previous studies have shown that many protein targets (80%–85% of the protein-coding genes) are still “undruggable”, mostly scaffold proteins or transcription factors.^130,131^ In contrast, 98% of the human transcriptome consist of noncoding RNAs; therefore, RNA therapy provides treatment options to those diseases with “undruggable” protein targets. Drug resistance from an ABC transporter or from epigenetic modifications is a serious issue in treating cancer or infectious disease,^132,133^ whereas ncRNA therapy has no such issues reported so far. Another advantage of ncRNA is the paracrine effect. Previous studies have shown that multiple cell types in the cardiovascular system generate different kinds of vesicles, such as microvesicles and exosomes, that are able to transport the ncRNAs to other organs or cell types. The paracrine effect provides ncRNA-based drugs with broader targets to the whole signaling pathway compared with antibodies or small molecules.^134,135^ Additionally, with different chemical modifications, the half-life of ncRNA drugs can be long (weeks to months), and, thus, patient dosing frequency can be decreased compared with small molecules or antibodies.^136,137^ A further advantage is that one or more complete disease pathway can be modulated by noncoding RNA-based therapeutic approaches.^138^

With respect to chemical modifications, the miRNA agonists and antagonists mentioned in the previous sections are all synthetic oligonucleotides but belong to different chemical classes. These range from small double-stranded RNAs (siRNA—eg, Patisiran and miRNA mimic—eg, MesomiR) over antisense DNA/RNA oligonucleotides with backbone modifications (eg, Fomivirsen, Eteplirsen). Furthermore, the second generation of antisense oligonucleotides (ASOs), which contain sugar modifications such as 2’-O-methoxyethyl (2’-O-MOE, eg, Mipomersen) or an 2’-4’ ether bridge leading to a bicyclic sugar moiety, usually referred to as LNA, have also been well-developed. To maintain RNAse H cleavage, these therapeutic ASOs need to possess a complementary sequence target made up of DNA flanked by the modified residues; these entities are called GapmeR or chimeric LNA (eg, Miravirsen, Cobomarsen; Figure 1).

The first steps in clinical development mainly used local administration, for example, intravitreal for eye diseases, intradermal for wound healing, and intrathecal for neurological disorders. Meanwhile, systemic administrations, such as intravenous or subcutaneous injection, are preferred for most clinical applications but raise the question of tissue-specific drug delivery versus off-target effects.^139^ Current nonclinical studies in heart disease models may make use of local administration, including intracoronary or intramyocardial injection, but their translation into clinical reality remains questionable and difficult (Figure 1). Therefore, different strategies have been exploited to direct therapeutic ASO to target tissue and cell types. In CVD, it may be important, depending on the pathomechanism of the clinical entity, to deliver the drug to the cardiomyocytes, endothelial cells, cardiac fibroblasts, or the immune cells in the heart. Viral and nonviral approaches are the subjects of ongoing investigations in RNA-based diagnostic and therapeutic strategies for CVD, as recently reviewed by Lu and Thum.^6^ While directing ASO drugs toward the liver via GalNAc conjugation or liposomal formulation has already reached a clinical stage, including Food and Drug Administration approval (Patisiran), microRNA mimics have been shown to specifically reach cancer cells via liposomal (MRX34) or TargomiR formulations (MesoMiR). However, the delivery of cardiac-specific ASO drug remains a challenge to be solved in the future (Figure 1).

In a systematic study involving 135 large animal pigs, potential differences between intravenous and intracoronary applications of antimiR-132 were tested.^23^ Based on detailed plasma PK and tissue uptake measurements of the antimiR-132, it could be shown that there were no significant PK differences between these 2 routes of administration. However, whether this could be translated to other antimiR molecules and/or chemistries remains to be tested.

Virus-based approaches, especially via adenoassociated virus, are currently powerful tools for human gene therapy but challenging due to high antibody titers found in many patients, which limits the number of eligible patients entering clinical trials.^140–143^ A possible solution could be new capsid-modified adenoassociated viruses with improved specificity for delivery to the cardiovascular system and/or a decreased ability to raise an immune response.^144–146^ Cardiac specific delivery strategies are also currently being developed in the ongoing EU project CardioReGenix.^147^

Finally, most studies have usually been performed in single-model systems in small rodents, hampering clinical translation. Cardiovascular studies in large-animal, nonrodent studies or human ex vivo studies may be more predictive of future clinical success in next generation ncRNA-based therapeutics.^97^

## Conclusions

**Figure 3.**
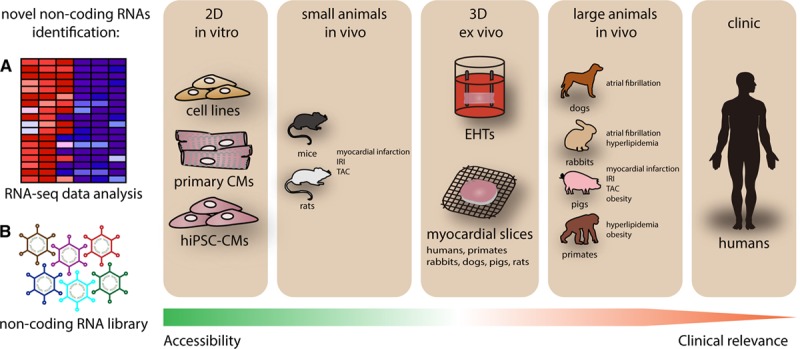
**Processes of noncoding RNA (ncRNA)-based drug development.** Novel ncRNA candidates are selected from a RNA-seq profile or other ncRNA approaches^40,148^ and then validated in cardiovascular cells (in vitro). After basic characterization, the ncRNA candidates are further investigated in animal models (in vivo). Several pathophysiological animal models of cardiovascular complication are available in different species, ranging from small to large animals, via the application of surgical techniques, genetic engineering, or diet changes. Some effective yet nontoxic ncRNA candidates are selected for further clinical development. However, successful transitions from preclinical to clinical studies are generally small in number. Often, in vitro models are easy to apply but have limited clinical relevance; while in vivo models have higher clinical relevance, they are challenging to conduct and expensive. To increase the translational efficiency of ncRNA-based therapeutic human induced pluripotent stem cells and/or other human cardiovascular cell types and living myocardial slices, could be powerful tools to bridge the gap between in vivo and clinical development. IRI indicates ischemia-reperfusion injury; and TAC, transverse aortic constriction.

Here, we described the state-of-the-art ncRNA-based therapies targeting the heart, ranging from large-animal heart disease models to clinical studies (Figure 3).

The improvement of bioinformatic tools enhances our understanding of the underlying mechanisms of the lncRNA/circRNA-mRNA-miRNA network in CVDs. This then supports the discovery of novel RNA molecules, which can prove to be therapeutic targets and provide more and hopefully improved treatment options to CVD patients in the future. Furthermore, large animal models have recently been gaining increasing attention for their high clinical relevance; however, while being closer to humans than rodents, there remain limitations on the level of metabolism or immune system, which render animal studies on a whole not to be fully predictive for safety and efficacy in humans. Despite great efforts in the last few decades, promising clinical candidates continue to be eliminated from the developmental pipeline for safety reasons and/or a lack of efficacy in all clinical stages. Therefore, the rapid development of human ex vivo systems, such as EHTs and living myocardial slices, constitute new valuable tools to improve insight into the translatability of preclinical studies. Such ex vivo models will also ultimately contribute to a reduction of the number of animals used in animal studies in pharmaceutical drug development. New delivery techniques with the aim of increasing tissue and/or cell type specificity and thereby lowering off-target effects will improve the safety of new developmental drugs. Moreover, an increased understanding of certain interindividual and sex differences is a requirement before progress in personalized medicine.

In addition to developing techniques in the laboratory, it is also important to validate and qualify these new tools and methods to achieve standardized assays that can be acceptable to authorities within the regulatory procedure.

In summary, based on (1) in vitro models, (2) rodent models, (3) large animal models, (4) ex vivo studies with human cells and tissues, and (5) new delivery systems, ncRNA therapies have the potential to enable significant progress in the development of next-generation therapeutics for cardiovascular disease (Figure 3).

## Acknowledgments

We thank M. Jung, S. Cushman, and S. Chatterjee (Institute of Molecular and Translational Therapeutic Strategies, Hannover Medical School, Hannover, Germany) for their help with the manuscript’s proofreading and their useful advice.

## Sources of Funding

This study was supported by the Leducq Foundation (via the MIRVAD consortium) and the European Union via an ERC grant to T. Thum (LONGHEART; EU H2020 GA 648038), the Deutsche Forschungsgemeinschaft (DFG Th903/19-1; TRR267, INST95/15641), and CardioReGenix (development of next-generation gene therapies for cardiovascular disease, EU H2020 GA 825670).

## Disclosures

T. Thum has filed and licensed patents regarding the diagnostic and therapeutic use of several cardiovascular noncoding RNAs. He is also the founder of and holds shares in Cardior Pharmaceuticals GmbH, a clinical-stage biotech company. The other authors report no conflicts.

## References

[1] Djebali S, Davis CA, Merkel A, Dobin A, Lassmann T, Mortazavi A, Tanzer A, Lagarde J, Lin W, Schlesinger F (2012). Landscape of transcription in human cells.. Nature.

[2] Salzman J, Gawad C, Wang PL, Lacayo N, Brown PO (2012). Circular RNAs are the predominant transcript isoform from hundreds of human genes in diverse cell types.. PLoS One.

[3] Andreassi MG (2018). Non-coding RNA in cardiovascular disease: a general overview on microRNAs, long non-coding RNAs and circular RNAs.. Non-coding RNA Invest.

[4] Poller W, Dimmeler S, Heymans S, Zeller T, Haas J, Karakas M, Leistner DM, Jakob P, Nakagawa S, Blankenberg S (2018). Non-coding RNAs in cardiovascular diseases: diagnostic and therapeutic perspectives.. Eur Heart J.

[5] Thum T (2014). Noncoding RNAs and myocardial fibrosis.. Nat Rev Cardiol.

[6] Lu D, Thum T (2019). RNA-based diagnostic and therapeutic strategies for cardiovascular disease.. Nat Rev Cardiol.

[7] Thum T, Gross C, Fiedler J, Fischer T, Kissler S, Bussen M, Galuppo P, Just S, Rottbauer W, Frantz S (2008). MicroRNA-21 contributes to myocardial disease by stimulating MAP kinase signalling in fibroblasts.. Nature.

[8] Viereck J, Kumarswamy R, Foinquinos A, Xiao K, Avramopoulos P, Kunz M, Dittrich M, Maetzig T, Zimmer K, Remke J (2016). Long noncoding RNA chast promotes cardiac remodeling.. Sci Transl Med.

[9] Timmis A, Townsend N, Gale C, Grobbee R, Maniadakis N, Flather M, Wilkins E, Wright L, Vos R, Bax J, ESC Scientific Document Group (2018). European Society of cardiology: cardiovascular disease statistics 2017.. Eur Heart J.

[10] Murphy SL, Xu J, Kochanek KD, Arias E (2018). Mortality in the United States, 2017.. NCHS data brief.

[11] Camacho P, Fan H, Liu Z, He JQ (2016). Large mammalian animal models of heart disease.. Journal of cardiovascular development and disease.

[12] Hearse DJ, Sutherland FJ (2000). Experimental models for the study of cardiovascular function and disease.. Pharmacol Res.

[13] Bennett CF, Baker BF, Pham N, Swayze E, Geary RS (2017). Pharmacology of antisense drugs.. Annu Rev Pharmacol Toxicol.

[14] Stein CA, Castanotto D (2017). FDA-approved oligonucleotide therapies in 2017.. Mol Ther.

[15] Al Shaer D, Al Musaimi O, Albericio F, de la Torre BG (2019). 2018 FDA tides harvest.. Pharmaceuticals.

[16] Beermann J, Piccoli MT, Viereck J, Thum T (2016). Non-coding RNAs in development and disease: background, mechanisms, and therapeutic approaches.. Physiol Rev.

[17] Gallant-Behm CL, Piper J, Dickinson BA, Dalby CM, Pestano LA, Jackson AL (2018). A synthetic microRNA-92a inhibitor (MRG-110) accelerates angiogenesis and wound healing in diabetic and nondiabetic wounds.. Wound Repair Regen.

[18] Hinkel R, Penzkofer D, Zühlke S, Fischer A, Husada W, Xu QF, Baloch E, van Rooij E, Zeiher AM, Kupatt C (2013). Inhibition of microRNA-92a protects against ischemia/reperfusion injury in a large-animal model.. Circulation.

[19] Bellera N, Barba I, Rodriguez-Sinovas A, Ferret E, Asín MA, Gonzalez-Alujas MT, Pérez-Rodon J, Esteves M, Fonseca C, Toran N (2014). Single intracoronary injection of encapsulated antagomir-92a promotes angiogenesis and prevents adverse infarct remodeling.. J Am Heart Assoc.

[20] Hullinger TG, Montgomery RL, Seto AG, Dickinson BA, Semus HM, Lynch JM, Dalby CM, Robinson K, Stack C, Latimer PA (2012). Inhibition of miR-15 protects against cardiac ischemic injury.. Circ Res.

[21] Eding JE, Demkes CJ, Lynch JM, Seto AG, Montgomery RL, Semus HM, Jackson AL, Isabelle M, Chimenti S, van Rooij E (2017). The efficacy of cardiac anti-miR-208a therapy is stress dependent.. Mol Ther.

[22] Gabisonia K, Prosdocimo G, Aquaro GD, Carlucci L, Zentilin L, Secco I, Ali H, Braga L, Gorgodze N, Bernini F (2019). MicroRNA therapy stimulates uncontrolled cardiac repair after myocardial infarction in pigs.. Nature.

[23] Foinquinos A, Batkai S, Genschel C, Viereck J, Rump S, Gyöngyösi M, Traxler D Preclinical development of a miR-132 inhibitor for heart failure treatment [published online January 31, 2020].. Nat Commun.

[24] Lu Y, Zhang Y, Wang N, Pan Z, Gao X, Zhang F, Zhang Y, Shan H, Luo X, Bai Y (2010). MicroRNA-328 contributes to adverse electrical remodeling in atrial fibrillation.. Circulation.

[25] Zhang Y, Zheng S, Geng Y, Xue J, Wang Z, Xie X, Wang J, Zhang S, Hou Y (2015). MicroRNA profiling of atrial fibrillation in canines: miR-206 modulates intrinsic cardiac autonomic nerve remodeling by regulating SOD1.. PLoS One.

[26] Jia X, Zheng S, Xie X, Zhang Y, Wang W, Wang Z, Zhang Y, Wang J, Gao M, Hou Y (2013). MicroRNA-1 accelerates the shortening of atrial effective refractory period by regulating KCNE1 and KCNB2 expression: an atrial tachypacing rabbit model.. PLoS One.

[27] Rayner KJ, Esau CC, Hussain FN, McDaniel AL, Marshall SM, van Gils JM, Ray TD, Sheedy FJ, Goedeke L, Liu X (2011). Inhibition of miR-33a/b in non-human primates raises plasma HDL and lowers VLDL triglycerides.. Nature.

[28] Rottiers V, Obad S, Petri A, McGarrah R, Lindholm MW, Black JC, Sinha S, Goody RJ, Lawrence MS, deLemos AS (2013). Pharmacological inhibition of a microRNA family in nonhuman primates by a seed-targeting 8-mer antimiR.. Sci Transl Med.

[29] Bonauer A, Carmona G, Iwasaki M, Mione M, Koyanagi M, Fischer A, Burchfield J, Fox H, Doebele C, Ohtani K (2009). MicroRNA-92a controls angiogenesis and functional recovery of ischemic tissues in mice.. Science.

[30] Nishi H, Ono K, Iwanaga Y, Horie T, Nagao K, Takemura G, Kinoshita M, Kuwabara Y, Mori RT, Hasegawa K (2010). MicroRNA-15b modulates cellular ATP levels and degenerates mitochondria via Arl2 in neonatal rat cardiac myocytes.. J Biol Chem.

[31] Eulalio A, Mano M, Dal Ferro M, Zentilin L, Sinagra G, Zacchigna S, Giacca M (2012). Functional screening identifies miRNAs inducing cardiac regeneration.. Nature.

[32] Montgomery RL, Hullinger TG, Semus HM, Dickinson BA, Seto AG, Lynch JM, Stack C, Latimer PA, Olson EN, van Rooij E (2011). Therapeutic inhibition of miR-208a improves cardiac function and survival during heart failure.. Circulation.

[33] Williams AH, Valdez G, Moresi V, Qi X, McAnally J, Elliott JL, Bassel-Duby R, Sanes JR, Olson EN (2009). MicroRNA-206 delays ALS progression and promotes regeneration of neuromuscular synapses in mice.. Science.

[34] Jeng SF, Rau CS, Liliang PC, Wu CJ, Lu TH, Chen YC, Lin CJ, Hsieh CH (2009). Profiling muscle-specific microRNA expression after peripheral denervation and reinnervation in a rat model.. J Neurotrauma.

[35] Terentyev D, Belevych AE, Terentyeva R, Martin MM, Malana GE, Kuhn DE, Abdellatif M, Feldman DS, Elton TS, Györke S (2009). miR-1 overexpression enhances Ca(2+) release and promotes cardiac arrhythmogenesis by targeting PP2A regulatory subunit B56alpha and causing CaMKII-dependent hyperphosphorylation of RyR2.. Circ Res.

[36] Yang B, Lin H, Xiao J, Lu Y, Luo X, Li B, Zhang Y, Xu C, Bai Y, Wang H (2007). The muscle-specific microRNA miR-1 regulates cardiac arrhythmogenic potential by targeting GJA1 and KCNJ2.. Nat Med.

[37] Rayner KJ, Suárez Y, Dávalos A, Parathath S, Fitzgerald ML, Tamehiro N, Fisher EA, Moore KJ, Fernández-Hernando C (2010). MiR-33 contributes to the regulation of cholesterol homeostasis.. Science.

[38] Najafi-Shoushtari SH, Kristo F, Li Y, Shioda T, Cohen DE, Gerszten RE, Näär AM (2010). MicroRNA-33 and the SREBP host genes cooperate to control cholesterol homeostasis.. Science.

[39] Rayner KJ, Sheedy FJ, Esau CC, Hussain FN, Temel RE, Parathath S, van Gils JM, Rayner AJ, Chang AN, Suarez Y (2011). Antagonism of miR-33 in mice promotes reverse cholesterol transport and regression of atherosclerosis.. J Clin Invest.

[40] Ucar A, Gupta SK, Fiedler J, Erikci E, Kardasinski M, Batkai S, Dangwal S, Kumarswamy R, Bang C, Holzmann A (2012). The miRNA-212/132 family regulates both cardiac hypertrophy and cardiomyocyte autophagy.. Nat Commun.

[41] Hennessy EJ, van Solingen C, Scacalossi KR, Ouimet M, Afonso MS, Prins J, Koelwyn GJ, Sharma M, Ramkhelawon B, Carpenter S (2019). The long noncoding RNA CHROME regulates cholesterol homeostasis in primate.. Nat Metab.

[42] Li DY, Busch A, Jin H, Chernogubova E, Pelisek J, Karlsson J, Sennblad B, Liu S, Lao S, Hofmann P (2018). H19 induces abdominal aortic aneurysm development and progression.. Circulation.

[43] Cui J, Li J, Mathison M, Tondato F, Mulkey SP, Micko C, Chronos NA, Robinson KA (2005). A clinically relevant large-animal model for evaluation of tissue-engineered cardiac surgical patch materials.. Cardiovasc Revasc Med.

[44] Clauss S, Bleyer C, Schüttler D, Tomsits P, Renner S, Klymiuk N, Wakili R, Massberg S, Wolf E, Kääb S (2019). Animal models of arrhythmia: classic electrophysiology to genetically modified large animals.. Nat Rev Cardiol.

[45] Meyers DE, Basha HI, Koenig MK (2013). Mitochondrial cardiomyopathy: pathophysiology, diagnosis, and management.. Tex Heart Inst J.

[46] van Rooij E, Sutherland LB, Qi X, Richardson JA, Hill J, Olson EN (2007). Control of stress-dependent cardiac growth and gene expression by a microRNA.. Science.

[47] Amuzie C, Swart JR, Rogers CS, Vihtelic T, Denham S, Mais DE (2016). A translational model for diet-related atherosclerosis: effect of statins on hypercholesterolemia and atherosclerosis in a minipig.. Toxicol Pathol.

[48] Fang B, Ren X, Wang Y, Li Z, Zhao L, Zhang M, Li C, Zhang Z, Chen L, Li X (2018). Apolipoprotein E deficiency accelerates atherosclerosis development in miniature pigs.. Dis Model Mech.

[49] Sewanan LR, Schwan J, Kluger J, Park J, Jacoby DL, Qyang Y, Campbell SG (2019). Extracellular matrix from hypertrophic myocardium provokes impaired twitch dynamics in healthy cardiomyocytes.. JACC Basic Transl Sci.

[50] Mentzel CM, Alkan F, Keinicke H, Jacobsen MJ, Gorodkin J, Fredholm M, Cirera S (2016). Joint profiling of miRNAs and mRNAs reveals miRNA mediated gene regulation in the göttingen minipig obesity model.. PLoS One.

[51] Bartunek J, Croissant JD, Wijns W, Gofflot S, de Lavareille A, Vanderheyden M, Kaluzhny Y, Mazouz N, Willemsen P, Penicka M (2007). Pretreatment of adult bone marrow mesenchymal stem cells with cardiomyogenic growth factors and repair of the chronically infarcted myocardium.. Am J Physiol Heart Circ Physiol.

[52] Brundel BJ, Melnyk P, Rivard L, Nattel S (2005). The pathology of atrial fibrillation in dogs.. J Vet Cardiol.

[53] Shan H, Zhang Y, Lu Y, Zhang Y, Pan Z, Cai B, Wang N, Li X, Feng T, Hong Y (2009). Downregulation of miR-133 and miR-590 contributes to nicotine-induced atrial remodelling in canines.. Cardiovasc Res.

[54] Shiomi M (2019). The history of the WHHL rabbit, an animal model of familial hypercholesterolemia (I) -contribution to the elucidation of the pathophysiology of human hypercholesterolemia and coronary heart disease.. J Atheroscler Thromb.

[55] Watanabe Y (1980). Serial inbreeding of rabbits with hereditary hyperlipidemia (WHHL-rabbit).. Atherosclerosis.

[56] Shen YT (2010). Primate models for cardiovascular drug research and development.. Curr Opin Investig Drugs.

[57] Sun X, Cai J, Fan X, Han P, Xie Y, Chen J, Xiao Y, Kang YJ (2013). Decreases in electrocardiographic R-wave amplitude and QT interval predict myocardial ischemic infarction in rhesus monkeys with left anterior descending artery ligation.. PLoS One.

[58] Brooks-Wilson A, Marcil M, Clee SM, Zhang LH, Roomp K, van Dam M, Yu L, Brewer C, Collins JA, Molhuizen HO (1999). Mutations in ABC1 in tangier disease and familial high-density lipoprotein deficiency.. Nat Genet.

[59] Horton JD, Goldstein JL, Brown MS (2002). SREBPs: activators of the complete program of cholesterol and fatty acid synthesis in the liver.. J Clin Invest.

[60] Bär C, Chatterjee S, Thum T (2016). Long noncoding RNAs in cardiovascular pathology, diagnosis, and therapy.. Circulation.

[61] Washietl S, Kellis M, Garber M (2014). Evolutionary dynamics and tissue specificity of human long noncoding RNAs in six mammals.. Genome Res.

[62] Necsulea A, Soumillon M, Warnefors M, Liechti A, Daish T, Zeller U, Baker JC, Grützner F, Kaessmann H (2014). The evolution of lncRNA repertoires and expression patterns in tetrapods.. Nature.

[63] Grote P, Wittler L, Hendrix D, Koch F, Währisch S, Beisaw A, Macura K, Bläss G, Kellis M, Werber M (2013). The tissue-specific lncRNA Fendrr is an essential regulator of heart and body wall development in the mouse.. Dev Cell.

[64] Liu L, An X, Li Z, Song Y, Li L, Zuo S, Liu N, Yang G, Wang H, Cheng X (2016). The H19 long noncoding RNA is a novel negative regulator of cardiomyocyte hypertrophy.. Cardiovasc Res.

[65] Capra JA, Singh M (2007). Predicting functionally important residues from sequence conservation.. Bioinformatics.

[66] Ponting CP (2017). Biological function in the twilight zone of sequence conservation.. BMC Biol.

[67] Kern C, Wang Y, Chitwood J, Korf I, Delany M, Cheng H, Medrano JF, Van Eenennaam AL, Ernst C, Ross P (2018). Genome-wide identification of tissue-specific long non-coding RNA in three farm animal species.. BMC Genomics.

[68] Wucher V, Legeai F, Hédan B, Rizk G, Lagoutte L, Leeb T, Jagannathan V, Cadieu E, David A, Lohi H (2017). FEELnc: a tool for long non-coding RNA annotation and its application to the dog transcriptome.. Nucleic Acids Res.

[69] Le Béguec C, Wucher V, Lagoutte L, Cadieu E, Botherel N, Hédan B, De Brito C, Guillory AS, André C, Derrien T (2018). Characterisation and functional predictions of canine long non-coding RNAs.. Sci Rep.

[70] Hezroni H, Koppstein D, Schwartz MG, Avrutin A, Bartel DP, Ulitsky I (2015). Principles of long noncoding RNA evolution derived from direct comparison of transcriptomes in 17 species.. Cell Rep.

[71] Sarropoulos I, Marin R, Cardoso-Moreira M, Kaessmann H (2019). Developmental dynamics of lncRNAs across mammalian organs and species.. Nature.

[72] Kaikkonen MU, Halonen P, Liu OH, Turunen TA, Pajula J, Moreau P, Selvarajan I, Tuomainen T, Aavik E, Tavi P (2017). Genome-wide dynamics of nascent noncoding RNA transcription in porcine heart after myocardial infarction.. Circulation Cardiovascular genetics.

[73] Wang WJ, Wang YM, Hu Y, Lin Q, Chen R, Liu H, Cao WZ, Zhu HF, Tong C, Li L (2018). HDncRNA: a comprehensive database of non-coding RNAs associated with heart diseases.. Database (Oxford).

[74] Wu Y, Zhang F, Li X, Hou W, Zhang S, Feng Y, Lu R, Ding Y, Sun L (2019). Systematic analysis of lncRNA expression profiles and atherosclerosis-associated lncRNA-mRNA network revealing functional lncRNAs in carotid atherosclerotic rabbit models.. Funct Integr Genomics.

[75] Cabili MN, Trapnell C, Goff L, Koziol M, Tazon-Vega B, Regev A, Rinn JL (2011). Integrative annotation of human large intergenic noncoding RNAs reveals global properties and specific subclasses.. Genes Dev.

[76] Santer L, Bär C, Thum T (2019). Circular RNAs: a novel class of functional RNA molecules with a therapeutic perspective.. Mol Ther.

[77] Sanger HL, Klotz G, Riesner D, Gross HJ, Kleinschmidt AK (1976). Viroids are single-stranded covalently closed circular RNA molecules existing as highly base-paired rod-like structures.. Proc Natl Acad Sci U S A.

[78] Aufiero S, Reckman YJ, Pinto YM, Creemers EE (2019). Circular RNAs open a new chapter in cardiovascular biology.. Nat Rev Cardiol.

[79] Wang K, Long B, Liu F, Wang JX, Liu CY, Zhao B, Zhou LY, Sun T, Wang M, Yu T (2016). A circular RNA protects the heart from pathological hypertrophy and heart failure by targeting miR-223.. Eur Heart J.

[80] Hansen TB, Jensen TI, Clausen BH, Bramsen JB, Finsen B, Damgaard CK, Kjems J (2013). Natural RNA circles function as efficient microRNA sponges.. Nature.

[81] Geng HH, Li R, Su YM, Xiao J, Pan M, Cai XX, Ji XP (2016). The circular RNA Cdr1as promotes myocardial infarction by mediating the regulation of miR-7a on its target genes expression.. PLoS One.

[82] Garikipati VNS, Verma SK, Cheng Z, Liang D, Truongcao MM, Cimini M, Yue Y, Huang G, Wang C, Benedict C (2019). Circular RNA CircFndc3b modulates cardiac repair after myocardial infarction via FUS/VEGF-A axis.. Nat Commun.

[83] Li Y, Zhang J, Huo C, Ding N, Li J, Xiao J, Lin X, Cai B, Zhang Y, Xu J (2017). Dynamic organization of lncRNA and circular RNA regulators collectively controlled cardiac differentiation in humans.. EBioMedicine.

[84] Lin F, Zhao G, Chen Z, Wang X, Lv F, Zhang Y, Yang X, Liang W, Cai R, Li J (2019). circRNA-miRNA association for coronary heart disease.. Mol Med Rep.

[85] Jiang S, Guo C, Zhang W, Che W, Zhang J, Zhuang S, Wang Y, Zhang Y, Liu B (2019). The integrative regulatory network of circRNA, microRNA, and mRNA in atrial fibrillation.. Front Genet.

[86] Zhang F, Zhang R, Zhang X, Wu Y, Li X, Zhang S, Hou W, Ding Y, Tian J, Sun L (2018). Comprehensive analysis of circRNA expression pattern and circRNA-miRNA-mRNA network in the pathogenesis of atherosclerosis in rabbits.. Aging (Albany NY).

[87] Kleaveland B, Shi CY, Stefano J, Bartel DP (2018). A network of noncoding regulatory RNAs acts in the mammalian brain.. Cell.

[88] Enuka Y, Lauriola M, Feldman ME, Sas-Chen A, Ulitsky I, Yarden Y (2016). Circular RNAs are long-lived and display only minimal early alterations in response to a growth factor.. Nucleic Acids Res.

[89] Holdt LM, Kohlmaier A, Teupser D (2018). Circular RNAs as therapeutic agents and targets.. Front Physiol.

[90] Jeck WR, Sorrentino JA, Wang K, Slevin MK, Burd CE, Liu J, Marzluff WF, Sharpless NE (2013). Circular RNAs are abundant, conserved, and associated with ALU repeats.. RNA.

[91] Rybak-Wolf A, Stottmeister C, Glažar P, Jens M, Pino N, Giusti S, Hanan M, Behm M, Bartok O, Ashwal-Fluss R (2015). Circular RNAs in the mammalian brain are highly abundant, conserved, and dynamically expressed.. Mol Cell.

[92] Venø MT, Hansen TB, Venø ST, Clausen BH, Grebing M, Finsen B, Holm IE, Kjems J (2015). Spatio-temporal regulation of circular RNA expression during porcine embryonic brain development.. Genome Biol.

[93] You X, Vlatkovic I, Babic A, Will T, Epstein I, Tushev G, Akbalik G, Wang M, Glock C, Quedenau C (2015). Neural circular RNAs are derived from synaptic genes and regulated by development and plasticity.. Nat Neurosci.

[94] Zaragoza C, Gomez-Guerrero C, Martin-Ventura JL, Blanco-Colio L, Lavin B, Mallavia B, Tarin C, Mas S, Ortiz A, Egido J (2011). Animal models of cardiovascular diseases.. J Biomed Biotechnol.

[95] Yang H, Wu Z (2018). Genome editing of pigs for agriculture and biomedicine.. Front Genet.

[96] Hirt MN, Hansen A, Eschenhagen T (2014). Cardiac tissue engineering: state of the art.. Circ Res.

[97] Perbellini F, Thum T (2019). Living myocardial slices: a novel multicellular model for cardiac translational research.. Eur Heart J.

[98] Takahashi K, Tanabe K, Ohnuki M, Narita M, Ichisaka T, Tomoda K, Yamanaka S (2007). Induction of pluripotent stem cells from adult human fibroblasts by defined factors.. Cell.

[99] Yu J, Vodyanik MA, Smuga-Otto K, Antosiewicz-Bourget J, Frane JL, Tian S, Nie J, Jonsdottir GA, Ruotti V, Stewart R (2007). Induced pluripotent stem cell lines derived from human somatic cells.. Science.

[100] Karakikes I, Ameen M, Termglinchan V, Wu JC (2015). Human induced pluripotent stem cell-derived cardiomyocytes: insights into molecular, cellular, and functional phenotypes.. Circ Res.

[101] Zhang J, Wilson GF, Soerens AG, Koonce CH, Yu J, Palecek SP, Thomson JA, Kamp TJ (2009). Functional cardiomyocytes derived from human induced pluripotent stem cells.. Circ Res.

[102] Gherghiceanu M, Barad L, Novak A, Reiter I, Itskovitz-Eldor J, Binah O, Popescu LM (2011). Cardiomyocytes derived from human embryonic and induced pluripotent stem cells: comparative ultrastructure.. J Cell Mol Med.

[103] Oikonomopoulos A, Kitani T, Wu JC (2018). Pluripotent stem cell-derived cardiomyocytes as a platform for cell therapy applications: progress and hurdles for clinical translation.. Mol Ther.

[104] Weinberger F, Mannhardt I, Eschenhagen T (2017). Engineering cardiac muscle tissue: a maturating field of research.. Circ Res.

[105] Tiburcy M, Hudson JE, Balfanz P, Schlick S, Meyer T, Chang Liao ML, Levent E, Raad F, Zeidler S, Wingender E (2017). Defined engineered human myocardium with advanced maturation for applications in heart failure modeling and repair.. Circulation.

[106] Mannhardt I, Breckwoldt K, Letuffe-Brenière D, Schaaf S, Schulz H, Neuber C, Benzin A, Werner T, Eder A, Schulze T (2016). Human engineered heart tissue: analysis of contractile force.. Stem Cell Reports.

[107] Prondzynski M, Lemoine MD, Zech AT, Horváth A, Di Mauro V, Koivumäki JT, Kresin N, Busch J, Krause T, Krämer E (2019). Disease modeling of a mutation in α-actinin 2 guides clinical therapy in hypertrophic cardiomyopathy.. EMBO Mol Med.

[108] Ou Q, Jacobson Z, Abouleisa RRE, Tang XL, Hindi SM, Kumar A, Ivey KN, Giridharan G, El-Baz A, Brittian K (2019). Physiological biomimetic culture system for pig and human heart slices.. Circ Res.

[109] Bussek A, Wettwer E, Christ T, Lohmann H, Camelliti P, Ravens U (2009). Tissue slices from adult mammalian hearts as a model for pharmacological drug testing.. Cell Physiol Biochem.

[110] Perbellini F, Watson SA, Scigliano M, Alayoubi S, Tkach S, Bardi I, Quaife N, Kane C, Dufton NP, Simon A (2018). Investigation of cardiac fibroblasts using myocardial slices.. Cardiovasc Res.

[111] Watson SA, Duff J, Bardi I, Zabielska M, Atanur SS, Jabbour RJ, Simon A, Tomas A, Smolenski RT, Harding SE (2019). Biomimetic electromechanical stimulation to maintain adult myocardial slices in vitro.. Nat Commun.

[112] Kang C, Qiao Y, Li G, Baechle K, Camelliti P, Rentschler S, Efimov IR (2016). Human organotypic cultured cardiac slices: new platform for High Throughput Preclinical Human Trials.. Sci Rep.

[113] Wang K, Lee P, Mirams GR, Sarathchandra P, Borg TK, Gavaghan DJ, Kohl P, Bollensdorff C (2015). Cardiac tissue slices: preparation, handling, and successful optical mapping.. Am J Physiol Heart Circ Physiol.

[114] Watson SA, Scigliano M, Bardi I, Ascione R, Terracciano CM, Perbellini F (2017). Preparation of viable adult ventricular myocardial slices from large and small mammals.. Nat Protoc.

[115] Ton VK, Mukherjee M, Judge DP (2014). Transthyretin cardiac amyloidosis: pathogenesis, treatments, and emerging role in heart failure with preserved ejection fraction.. Clin Med Insights Cardiol.

[116] Jarvis LM (2019). The new drugs of 2018.. Chem Engineer News.

[117] Büller HR, Bethune C, Bhanot S, Gailani D, Monia BP, Raskob GE, Segers A, Verhamme P, Weitz JI, FXI-ASO TKA Investigators (2015). Factor XI antisense oligonucleotide for prevention of venous thrombosis.. N Engl J Med.

[118] Graham MJ, Lee RG, Brandt TA, Tai LJ, Fu W, Peralta R, Yu R, Hurh E, Paz E, McEvoy BW (2017). Cardiovascular and metabolic effects of ANGPTL3 antisense oligonucleotides.. N Engl J Med.

[119] Tsimikas S, Viney NJ, Hughes SG, Singleton W, Graham MJ, Baker BF, Burkey JL, Yang Q, Marcovina SM, Geary RS (2015). Antisense therapy targeting apolipoprotein(a): a randomised, double-blind, placebo-controlled phase 1 study.. Lancet.

[120] Deng Z, He Y, Yang X, Shi H, Shi A, Lu L, He L (2017). MicroRNA-29: a crucial player in fibrotic disease.. Mol Diagn Ther.

[121] van Rooij E, Sutherland LB, Thatcher JE, DiMaio JM, Naseem RH, Marshall WS, Hill JA, Olson EN (2008). Dysregulation of microRNAs after myocardial infarction reveals a role of miR-29 in cardiac fibrosis.. Proc Natl Acad Sci U S A.

[122] Sassi Y, Avramopoulos P, Ramanujam D, Grüter L, Werfel S, Giosele S, Brunner AD, Esfandyari D, Papadopoulou AS, De Strooper B (2017). Cardiac myocyte miR-29 promotes pathological remodeling of the heart by activating Wnt signaling.. Nat Commun.

[123] Gallant-Behm CL, Piper J, Lynch JM, Seto AG, Hong SJ, Mustoe TA, Maari C, Pestano LA, Dalby CM, Jackson AL (2019). A microRNA-29 mimic (Remlarsen) represses extracellular matrix expression and fibroplasia in the skin.. J Invest Dermatol.

[124] Kashtan C (2017). Alport syndrome: facts and opinions.. F1000Res.

[125] Guo J, Song W, Boulanger J, Xu EY, Wang F, Zhang Y, He Q, Wang S, Yang L, Pryce C (2019). Dysregulated expression of microRNA-21 and disease-related genes in human patients and in a mouse model of alport syndrome.. Hum Gene Ther.

[126] Gomez IG, MacKenna DA, Johnson BG, Kaimal V, Roach AM, Ren S, Nakagawa N, Xin C, Newitt R, Pandya S (2015). Anti-microRNA-21 oligonucleotides prevent alport nephropathy progression by stimulating metabolic pathways.. J Clin Invest.

[127] Heymans S, Corsten MF, Verhesen W, Carai P, van Leeuwen RE, Custers K, Peters T, Hazebroek M, Stöger L, Wijnands E (2013). Macrophage microRNA-155 promotes cardiac hypertrophy and failure.. Circulation.

[128] van Zandwijk N, Pavlakis N, Kao SC, Linton A, Boyer MJ, Clarke S, Huynh Y, Chrzanowska A, Fulham MJ, Bailey DL (2017). Safety and activity of microRNA-loaded minicells in patients with recurrent malignant pleural mesothelioma: a first-in-man, phase 1, open-label, dose-escalation study.. Lancet Oncol.

[129] Frazier KS (2015). Antisense oligonucleotide therapies: the promise and the challenges from a toxicologic pathologist’s perspective.. Toxicol Pathol.

[130] Russ AP, Lampel S (2005). The druggable genome: an update.. Drug Discov Today.

[131] Salami J, Crews CM (2017). Waste disposal-an attractive strategy for cancer therapy.. Science.

[132] Brown R, Curry E, Magnani L, Wilhelm-Benartzi CS, Borley J (2014). Poised epigenetic states and acquired drug resistance in cancer.. Nat Rev Cancer.

[133] Choi YH, Yu AM (2014). ABC transporters in multidrug resistance and pharmacokinetics, and strategies for drug development.. Curr Pharm Des.

[134] Thum T, Condorelli G (2015). Long noncoding RNAs and microRNAs in cardiovascular pathophysiology.. Circ Res.

[135] Loyer X, Vion AC, Tedgui A, Boulanger CM (2014). Microvesicles as cell-cell messengers in cardiovascular diseases.. Circ Res.

[136] Geary RS, Norris D, Yu R, Bennett CF (2015). Pharmacokinetics, biodistribution and cell uptake of antisense oligonucleotides.. Adv Drug Deliv Rev.

[137] Yin W, Rogge M (2019). Targeting RNA: a transformative therapeutic strategy.. Clin Transl Sci.

[138] Kreutzer FP, Fiedler J, Thum T (2019). Non-coding RNAs: key players in cardiac disease.. J Physiol.

[139] Shen X, Corey DR (2018). Chemistry, mechanism and clinical status of antisense oligonucleotides and duplex RNAs.. Nucleic Acids Res.

[140] Calcedo R, Wilson JM (2013). Humoral immune response to AAV.. Front Immunol.

[141] Calcedo R, Morizono H, Wang L, McCarter R, He J, Jones D, Batshaw ML, Wilson JM (2011). Adeno-associated virus antibody profiles in newborns, children, and adolescents.. Clin Vaccine Immunol.

[142] Hajjar RJ, Ishikawa K (2017). Introducing genes to the heart: all about delivery.. Circ Res.

[143] Mingozzi F, Büning H (2015). Adeno-associated viral vectors at the frontier between tolerance and immunity.. Front Immunol.

[144] Hammoudi N, Ishikawa K, Hajjar RJ (2015). Adeno-associated virus-mediated gene therapy in cardiovascular disease.. Curr Opin Cardiol.

[145] Ishikawa K, Fish KM, Tilemann L, Rapti K, Aguero J, Santos-Gallego CG, Lee A, Karakikes I, Xie C, Akar FG (2014). Cardiac I-1c overexpression with reengineered AAV improves cardiac function in swine ischemic heart failure.. Mol Ther.

[146] Mingozzi F, Anguela XM, Pavani G, Chen Y, Davidson RJ, Hui DJ, Yazicioglu M, Elkouby L, Hinderer CJ, Faella A (2013). Overcoming preexisting humoral immunity to AAV using capsid decoys.. Sci Transl Med.

[147] CardioReGenix Development of Next-Generation Gene Therapies for Cardiovascular Disease..

[148] Beermann J, Kirste D, Iwanov K, Lu D, Kleemiß F, Kumarswamy R, Schimmel K, Bär C, Thum T (2018). A large shRNA library approach identifies lncRNA Ntep as an essential regulator of cell proliferation.. Cell Death Differ.

